# Relative safety of glyphosate-resistant maize (CC-2) in rats

**DOI:** 10.1080/21645698.2025.2550820

**Published:** 2025-09-12

**Authors:** Anyi Liu, Xueqian Yin, Jing Wen, Chao Hou, Ruoyu Zhou, Xinran Liu, Ning Yin, Yuanzhi Jian, Shan Liu, Xiaoxuan Zhang, Junbo Wang

**Affiliations:** a Department of Nutrition and Food Hygiene, School of Public Health, Peking University, Beijing, China; b Key Laboratory of Food Safety Risk Assessment of Ministry of Health, China National Center for Food Safety Risk Assessment, Beijing, China; c Beijing Key Laboratory of Toxicological Research and Risk Assessment for Food Safety, Peking University, Beijing, China

**Keywords:** Glyphosate-Resistant Maize, genetically modified

## Abstract

With the rapid adoption of glyphosate-resistant (GR) crops by farmers, the food safety has garnered significant attention. This study aims to evaluate the relative safety of glyphosate-resistant maize (CC-2). Rats were divided into three groups: one group receiving AIN-93 G feed (AIN), one receiving non-modified receptor maize feed (NM), and the other receiving CC-2 maize feed (GM). The intervention spanned from gestation d 0 of the parental rats to 90 d post-weaning of the offspring. The results indicated no significant differences in pregnancy outcomes, body weight, growth monitoring, behavioral tests, or organ indexes between the GM group and the two control groups (AIN and NM). Although there were significant differences in some hematological parameters, serum chemistry, and organ weights, histopathological analysis revealed no abnormalities. No exogenous gene fragments were detected in major organs. In conclusion, CC-2 maize is relative safe for growth and development in rats.

## Introduction

As society continues to develop, humanity faces escalating challenges, including the depletion of natural resources, explosive population growth, pests, crop diseases, and limitations in both conventional and modern breeding methods.^1,2^ These challenges have spurred the development of new technologies aimed at overcoming these obstacles. Genetically modified (GM) crops have emerged as a significant innovation, effectively addressing these challenges and offering numerous benefits to growers worldwide.^2^ The key benefits include agronomic and economic advantages, modifications in the chemical composition of food, improvements in food processing, and the development of products for therapeutic purposes.^1^

Since their commercial introduction in 1994, GM crops have been rapidly adopted globally due to their substantial growth potential. A diverse range of crops with stable and novel genetic traits have been developed and commercialized, including corn, soybean, cotton, canola, potato, papaya, alfalfa, pumpkin, and sugar beet.^3^ Glyphosate-resistant (GR) crops, in particular, represent a class of genetically engineered products developed in response to the extensive use of glyphosate herbicides.^4^ Glyphosate herbicides, which specifically target 5-enolpyruvyl shikimate-3-phosphate synthase (EPSPS), are renowned for their broad spectrum, high efficiency, and low residue, making them the most widely used herbicides globally.^5^

Traditional crops also contain EPSPS targets, meaning glyphosate application during crop growth adversely affects crop development while eliminating weeds. Genetic engineering has enabled the development of crops resistant to glyphosate, allowing for more cost-effective and efficient weed control. Consequently, GR crops have been rapidly embraced by agricultural producers, representing the fastest adopted GM crop in agricultural history.^6^

Despite their benefits, public concerns regarding the food safety of GM crops persists. These concerns may stem from three potential sources: the inserted genes and their expressed proteins, secondary or pleiotropic effects of gene expression products, and possible disruption of natural genes in the recipient organism.^1,7^ Food safety assessments typically combine three approaches: investigation of structure/function relationships for indications of potential toxicity and allergenicity, in vitro assays using enzymes, receptor proteins, or cultured cell lines, and in vivo animal studies.^8^ Among these approaches, animal studies often provide the most indicative evidence of the potentially toxic effects of a test substance in humans.^8^

Current animal experiments evaluating the overall food safety of GR crops primarily focus on sub chronic^9^ and chronic toxicity,^10^ with relatively few studies examining toxicity during specific physiological stages. Therefore, this study was designed to evaluate the relative safety of GR crops used as feeds in rats based on guidelines and previous research. The crop used in this study is CC-2 maize, developed by China Agricultural University (CAU). Researchers at CAU cloned the Sorghum bicolor chloroplast transit peptide (CTP) of the EPSPS gene, fused it with the Agrobacterium sp. strain CP4 EPSPS gene, and obtained the maroACC gene.^11^ This gene expresses the maroACC protein, which confers glyphosate resistance to crops.

The study trial was informed by several guidelines: the Chinese Standard for Extended One-Generation Reproductive Toxicity Study^12^ the Chinese Standard for the 90-d Oral Toxicity Study,^13^ the OECD Guideline for the Extended One-Generation Reproductive Toxicity Study,^14^ the OECD Guideline for the Developmental Neurotoxicity Study,^15^ and the United States Environmental Protection Agency Guideline for the Prenatal Developmental Toxicity Study.^16^ The intervention began on gestation d 0 to focus on the growth and development of the offspring. Intervening 2 weeks before mating, common in reproductive developmental toxicity studies, could affect parental ova and sperm, which we aimed to avoid in this study.

Previous whole food safety assessments of maroACC transgenic crops^17–21^ revealed few significant adverse effects, leading us to use the maximum dose to observe more pronounced intervention effects. Following the EFSA guidance for GM organism risk assessment, we used a single dose level of GM test material at the maximum incorporation rate.^22^ To mitigate potential effects of high corn doses in animal feed due to different nutrient sources,^23^ we included the AIN-93 G feed group as a blank control to establish a standard growth curve and health status for the rats.

Given the previous safety assessments’ general results, we reduced the sample size according to safety assessment guidelines. Due to insufficient prior information, power analysis was challenging, so we determined the sample size for offspring to be eight animals per group based on literature^9,24^ and reduced the parental sample size proportionately. Using the “resource equation,”^25^ we verified that this sample size met the experimental requirements.

## Materials and Methods

### Plant Materials and Feeds

GR maize (CC-2) was used as the intervention, with non-transgenic maize (Dongdan 119, DD-119) serving as the control. Both types of maize were provided by CAU. The nutritional components of the two maize varieties were analyzed by the Beijing Institute of Nutrition Sources (National Laboratory Certification CNAS No. L2678; Qualification Certificate No. 150100340027). To ensure the feed contained as much carbohydrate as possible, the supplemental amount of CC-2 maize in the feed was 85.92%, based on the nutritional analysis and the AIN-93 G feed formula. Consequently, three diets were designed with theoretically equal concentrations of protein, carbohydrate, and fat as shown in Table 1.

**Table 1.** Formulae of feeds.

Ingredients (%)	AIN-93G feed	Non-transgenic feed	transgenic feed
Maize	0.00	85.92 (DD-119)	85.92 (CC-2)
Corn starch^*a*^	39.75	0.94	0.00
Dextrin	13.20	0.50	0.50
Sucrose	10.00	0.00	0.00
Cellulose	5.00	0.00	0.00
Casein^*b*^	20.00	11.62	11.82
Soybean oil	7.00	3.45	3.28
Mineral mix	3.50	3.50	3.50
Vitamin mix	1.08	1.08	1.08
L-Cystine	0.324	0.324	0.324
Choline Bitartrate	0.25	0.25	0.25
t-Butylhydroquinone	0.0014	0.0014	0.0014

^a^The moisture content of corn starch was 8.18%, b: the purity of casein was 93.50%.

The three feeds were prepared by Beijing Xiaoshuyoutai Biotechnology Co., Ltd. Animal feed license number: SCXK (Beijing) 2018–0006. The specific formulas of the three feeds are detailed in Table 1.

### Animals and Bioethics

Forty-eight female SD rats (weighing 230–280 g, gestation d 0) were provided by the Department of Laboratory Animal Science, Peking University Health Science Center (Animal certificate No.: SCXK11–00–0004; Laboratory Animal Production Licence No.: SCXK (Beijing) 2016–0010; Laboratory Animal Use License No.: SCXK (Beijing) 2016–0041). The rats were housed in a specific pathogen-free (SPF) animal feeding room of the Department of Laboratory Animal Science, Peking University Health Science Center (Animal Experimental Barrier Environment Qualification Certificate: Yidongzi No. 01–2055). Pregnant rats were housed individually, and four rats of the same sex lived together after weaning. Rats were given ad libitum access to irradiation sterilized feed and water. The temperature of the animal feeding room was 24°C–26°C, the relative humidity was 50%–60%, and indoor lighting was a 12 h light/dark cycle. Animal feeding, management, and experimental operation were carried out in accordance with the Regulations of Beijing on the Administration of Experimental Animals. The study protocol was reviewed by the Ethics Committee of Peking University Health Science Center (Ethics Approval No. LA2020438).

### Experimental Trial

Pregnant rats (parental generation) were randomly divided into three groups (*n* = 16 per group) and fed different feeds from gestation d 0. The three feed groups including the blank control group (AIN) which were fed with AIN-93 G; normal control group (NM) which were fed non-transgenic feed (85.92% DD-119 maize) and genetically modified group (GM) which were fed with 85.92% CC-2 maize. From gestation d 0, the paternal rats were monitored daily, and the pregnancy outcome indicators were recorded after delivery. The offspring rats were monitored daily from birth, and the litter was standardized on postnatal d 4 (PND 4) with four female and four male rats retained in each litter.

Rats were weaned on PND 21. Post-weaning, eight female and eight male rats from each group were randomly retained for a 90-d feeding experiment (PND 21–111), receiving the same feed as their mothers. At the end of the experiment, offspring were fasted overnight and then euthanized under anesthesia combined with exsanguination, with subsequent collection of blood and organs.

### Experimental Indicators

#### Pregnancy Outcomes

After delivery, the number of live pups, stillbirths, female pups, and male pups in each litter were counted, and the sex ratio (female/male) was calculated. The number of live pups on PND 4 was recorded to calculate the birth survival rate (the number of live pups on PND 4/liveborn number), while the number of live pups on PND 21 was recorded to calculate the lactation survival rate (the number of live pups on PND 21/the number of live pups on PND 4).

#### Body Weight and Food Consumption

On PND 0, 4, 7, 14, and 21, pups were weighed to draw the body weight change curve of during-lactation body weight. Post-weaning, body weight, and food consumption were recorded weekly. The feed conversion ratio (FCR) was calculated using the following equation.^26,27^

FCR = feed intake/bodyweight gain

#### Growth Monitoring and Tests

Since PND 0, the litter as a unit was observed daily to monitor physiological development and testing behavioral development. The monitoring and tests schedule are shown in Tables 2 and 3.

**Table 2.** Physiological development monitoring schedule.

Item	Milestone/method	Start	End
Anogenital distance (mm)	Distance from the anus to the genitalia	PND 4	PND 4
Auricle separation (day)	Both ears are separated from the outer skin of the head	PND 1	Each pup reach developmental milestones
Eye opening (day)	Both eyes open	PND 8	
Incisor eruption (day)	both upper and lower incisors erupt	PND 9	
Hair growth (day)	The skin all over the body grows white soft hair	PND 12

**Table 3.** Behavioral tests schedule during lactation.

Item	Initial state	Milestone/method	Start	End
Cliff avoidance (day)	The tip of the nose and forelimbs are exposed outside the edge of the platform	Turn or retreat to the platform within 30 s	PND 2	Each pup reach developmental milestones
Negative geotaxis (day)	Be placed head downward on a 30° inclined surface	Turn 180° upward in 30 s	PND 2	
Surface righting (day)	Lie on a platform with four limbs up	Turn over and land on three feet within 2 s for three consecutive tests	PND 4	
Air righting (day)	Fall with four limbs up	Turn over in the air and land on three feet for three consecutive tests	PND 12	
Forelimb hanging (s)	Hang from a 2 mm diameter wire by the forelimb alone	The duration of hanging before falling is recorded, keeping the maximum value from three consecutive tests.	PND 14	PND 14

After weaning, the rats were subjected to open field tests at PND 21 and 104. The tests used a black open field box of 100 cm × 100 cm × 50 cm, and the bottom of the box was evenly divided into 25 squares (5 × 5) by white thin tape. One rat was placed from the center each time, and the behavior was observed and recorded for 180 s.

Morris water maze tests were conducted on PND 22–27 and 105–110 using a cylindrical black pool (160 cm diameter, 50 cm depth). The water in the pool with a depth of 35 cm was dyed with 250 g of black ink and was maintained at 22°C. The pool was divided into four quadrants. A black circular platform with a diameter of 12 cm was placed 2 cm below the water surface in quadrant II.

Each test lasted for 6 d: hidden platform training trial was conducted on the first 5 d, the time rats spent to find the platform was recorded (4 times a day, no more than 1 min each time). On the 6^th^ d, the platform was removed, and the exploratory behavior of the rats was recorded within 1 min.

#### Hematology and Serum Biochemistry

An automatic hematology analyzer was used to analyze blood routine indicators, including white blood cell (WBC), red blood cell (RBC), hemoglobin (HGB), hematocrit (HCT), mean corpuscular volume (MCV), mean corpuscular hemoglobin (MCH), mean corpuscular hemoglobin concentration (MCHC), red blood cell distribution width (RDW), platelet count (PLT), platelet hematocrit (PCT), mean platelet volume (MPV), platelet distribution width (PDW), lymphocyte count (LYM), monocyte count (MONO), granulocyte count (GRN), lymphocyte percentage (LYM%), monocyte percentage (MONO%) and granulocyte percentage (GRN%).

An automatic serum biochemical analyzer was used to analyze serum biochemical indicators, including alanine aminotransferase (ALT), aspartate aminotransferase (AST), creatine kinase (CK), lactate dehydrogenase (LDH), alkaline phosphatase (ALP), total protein (TP), albumin (ALB), total bilirubin (TBIL), creatinine (CR), blood urea nitrogen (BUN), blood glucose (GLU), total cholesterol (TC), triglyceride (TG), high-density lipoprotein cholesterol (HDL-C), and low-density lipoprotein cholesterol (LDL-C).

#### Organ Weight and Histopathology

After the rats were sacrificed, major organs were weighed, and the organ indexes were calculated. Organs included the brain, heart, liver, spleen, thymus, kidneys, adrenal glands, ovaries, uteri, epididymis, and testis.

The liver, kidney, spleen, uterus, ovary, testis, and duodenum of three female rats and three male rats were randomly selected in each group. The above organs were fixed with 10% neutral formaldehyde solution, embedded in paraffin, sectioned, and stained with hematoxylin–eosin (H&E). Histopathological changes were observed under the microscope.

#### Maroacc Gene Detection

The brain, liver, kidney, spleen, uterus, and testis of three female rats and three male rats were randomly selected from the NM group and the GM group. The presence of the maroACC gene in the above organs was assessed by polymerase chain reaction (PCR). The PCR primers of the detected exogenous genes and animal reference genes are shown in Table 4.

**Table 4.** Qualitative PCR primers.

Primer name	Orientation	Primer sequence (5’ − 3’)	Amplicon size (bp)
maroACC	sense	gtgtccgagaaccctgtg	188
	anti-sense	ccttatctgggaactactcac	
β-actin	sense	tgttcgaagcaggaagatgac	209
	anti-sense	ttcctacaagcttttcccgc	

### Statistical Analyses

Data analysis was conducted using Excel 2016 and SPSS 23.0, with results expressed as the mean  ±  SD. A normality test and the homogeneity test of variance were carried out in advance. For normally distributed data, if the variance of data was homogeneous, one-way ANOVA was used for intergroup comparison; otherwise, Welch’s test was used. A rank-sum test was used for non-normally distributed data. Statistical significance was set at *p* < .05.

## Results

### Nutritional Component of Maize

The analysis of energy and main nutrients revealed no significant differences in energy, protein, fat, carbohydrate, fiber, water, and ash between CC-2 and DD-119 maize (Table 5).

**Table 5.** Nutritional components of maize (/100 g, *n* = 3).

component	CC-2	DD-119
energy (kcal)	367.03 ± 1.08	361.62 ± 3.32
protein (g)	8.90 ± 0.06	9.12 ± 0.18
fat (g)	4.33 ± 0.06	4.13 ± 0.12
carbohydrate (g)	68.90 ± 0.10	67.90 ± 0.92
fibre (g)	6.59 ± 0.25	6.34 ± 0.32
ash (g)	1.17 ± 0.06	1.23 ± 0.06
moisture (g)	10.13 ± 0.32	11.27 ± 1.10

### Pregnancy Outcome

There were no significant differences in gestational age, pup weight, litter weight, liveborn number, number of female and male pups, sex ratio, birth survival rate, and lactation survival rate among the three groups (Table 6).

**Table 6.** Litter observations (*n* = 16).

	AIN	NM	GM
litter weight (g)	83.94 ± 15.57	91.75 ± 13.10	88.16 ± 12.56
pup weight (g)	6.82 ± 0.76	7.14 ± 0.38	7.24 ± 0.50
gestational age (day)	21.75 ± 0.45	21.94 ± 0.25	22.00 ± 0.52
liveborn (No.)	12.38 ± 2.25	12.88 ± 1.93	12.25 ± 1.95
female pup (No.)	6.81 ± 1.83	6.25 ± 1.91	6.88 ± 1.54
male pup (No.)	5.56 ± 1.86	6.63 ± 1.75	5.38 ± 1.82
sex ratio	0.89 ± 0.41	1.20 ± 0.57	0.86 ± 0.52
birth survival rate (%)	99.58 ± 1.67	98.08 ± 7.69	98.41 ± 3.45
lactation survival rate (%)	99.22 ± 3.13	99.22 ± 3.13	100.00 ± 0.00

### Body Weight

**Figure 1.**
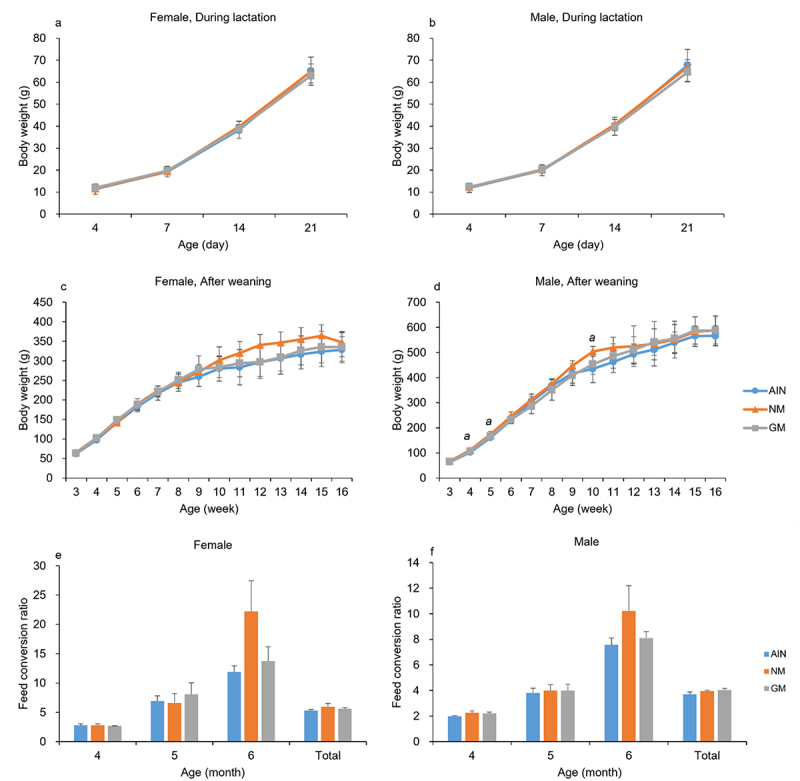
Body weight (during lactation, *n*=16; after weaning, *n*=8) and feed conversion ratio (*n*=2).

During lactation, there were no significant differences in body weight among all groups. Post-weaning, no significant differences were observed in female rats’ body weights among groups. However, male rats in the NM group at 4, 5, and 10 weeks had higher body weights than those in the AIN group (*p* < .05). The body weight changes are illustrated in Figure 1, while detailed data are provided in a supplementary document (Table S1). In the 6th month, the feed conversion ratio of the NM group was higher than that of the other two groups, although the difference was not significant (Figure 1, Table S2).

***a***: *P* < 0.05 significantly different from the AIN group.

### Growth Monitoring and Tests

#### Physiological and Behavioral Development During Lactation

**Figure 2.**
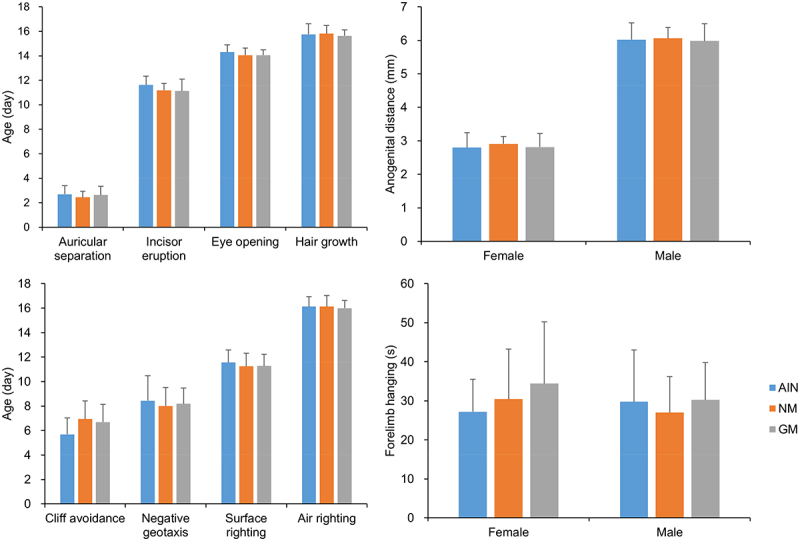
Physiological and behavioral development during lactation (*n*=16).

The results of physiological development monitoring and behavioral tests showed that there were no significant differences among the groups (Figure 2).

#### Open Field Tests

**Figure 3.**
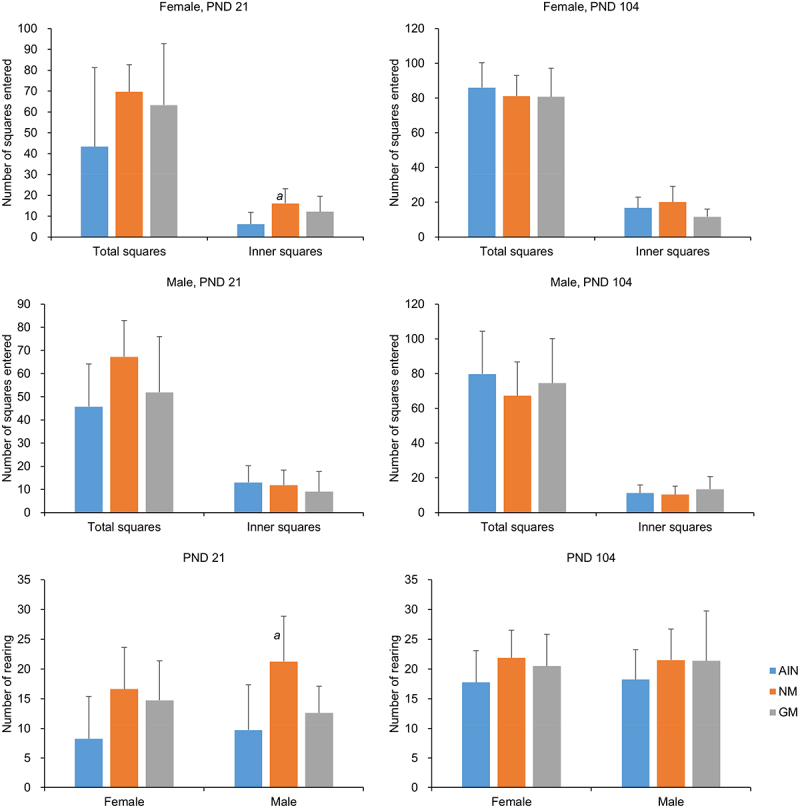
The open field test performance in rats (*n*=8).

Figure 3 presents the results of the two open field tests. In the first open field test (PND 21), the NM group exhibited a higher number of crossings inner squares and rearing times compared to the AIN group (p < .05). No significant differences were found in other indicators among the three groups.

***a***: P < .05 significantly different from the AIN group.

#### Morris Water Maze Tests

**Figure 4.**
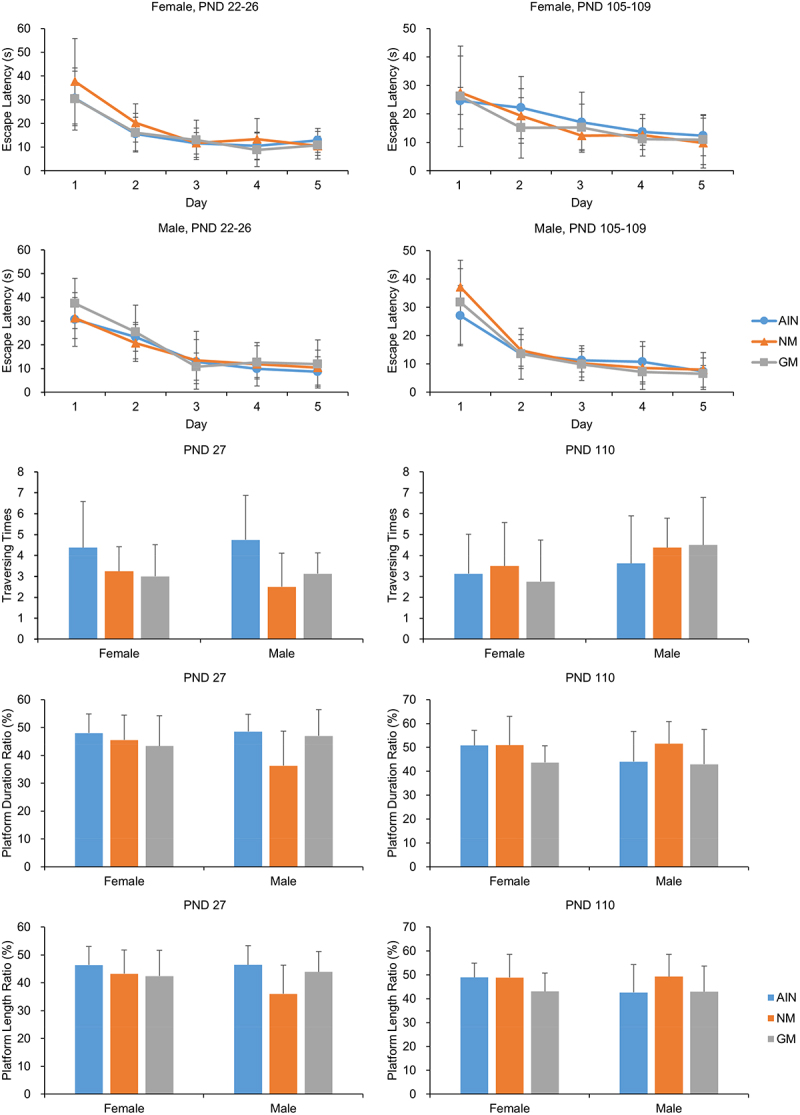
Morris water maze performance in rats (*n*=8).

As shown in Figure 4, there were no significant differences in the daily latency of the first 5 d or the exploration behaviors of the 6^th^ d among the groups in the two Morris water maze tests.

### Hematology and Serum Biochemistry

Hematology results (Table 7) indicated that male rats in the NM group had higher PLT, PCT, and MON% values than those in the AIN group (*p* < .05), and the MPV of male rats in the GM group was higher than that in the AIN group (*p* < .05). There were no significant differences in other blood routine indicators among the three groups.

**Table 7.** Hematology in rats (n = 8).

	female			male		
	AIN	NM	GM	AIN	NM	GM
WBC (10^9^/l)	6.81 ± 2.32	8.33 ± 3.34	7.08 ± 2.78	11.16 ± 1.95	10.78 ± 2.18	11.78 ± 3.88
RBC (10^12^/l)	7.95 ± 0.68	8.32 ± 0.55	7.45 ± 2.37	9.00 ± 0.32	9.12 ± 0.50	8.87 ± 1.02
HGB (g/l)	166.38 ± 13.95	175.25 ± 8.26	158.13 ± 51.82	180.38 ± 6.12	179.88 ± 12.3	172.75 ± 16.68
HCT (%)	43.50 ± 3.67	45.80 ± 2.61	41.70 ± 13.30	48.74 ± 1.77	47.98 ± 2.54	47.19 ± 4.60
MCV (fl)	54.70 ± 0.65	55.10 ± 2.01	55.88 ± 1.17	54.13 ± 0.92	52.55 ± 1.25	53.34 ± 1.42
MCH (pg)	20.91 ± 0.68	21.11 ± 1.10	21.00 ± 0.98	20.01 ± 0.68	19.69 ± 0.84	19.55 ± 0.80
MCHC (g/l)	382.75 ± 12.09	382.75 ± 8.17	375.50 ± 13.95	370.25 ± 10.86	374.75 ± 11.26	366.13 ± 9.30
RDW (%)	13.70 ± 0.49	13.94 ± 0.52	13.65 ± 0.60	14.58 ± 0.69	14.99 ± 0.87	14.61 ± 0.36
PLT (10^9^/l)	425.50 ± 157.10	473.00 ± 81.72	491.88 ± 170.70	442.25 ± 54.97	565.00 ± 83.71^*a*^	438.63 ± 247.03
PCT (%)	0.33 ± 0.12	0.37 ± 0.08	0.38 ± 0.13	0.33 ± 0.04	0.44 ± 0.07^*a*^	0.34 ± 0.19
MPV (fl)	7.84 ± 0.18	7.90 ± 0.47	7.80 ± 0.32	7.60 ± 0.14	7.95 ± 0.35	8.10 ± 0.48^*a*^
PDW (%)	12.39 ± 0.51	12.61 ± 0.58	12.83 ± 0.31	12.40 ± 0.56	12.39 ± 0.29	12.69 ± 0.29
LYM (10^9^/l)	4.56 ± 1.79	4.95 ± 1.14	4.91 ± 2.15	8.54 ± 1.98	7.55 ± 1.84	8.54 ± 3.43
MON (10^9^/l)	0.78 ± 0.30	0.91 ± 0.16	0.81 ± 0.27	1.41 ± 0.34	1.86 ± 0.57	1.91 ± 0.93
GRN (10^9^/l)	1.48 ± 0.57	2.46 ± 3.30	1.35 ± 0.94	1.21 ± 0.66	1.36 ± 0.67	1.33 ± 0.79
LYM% (%)	65.58 ± 7.21	64.19 ± 17.68	66.88 ± 11.19	75.69 ± 6.04	69.44 ± 6.79	71.10 ± 7.36
MON% (%)	11.29 ± 2.27	11.64 ± 2.96	12.36 ± 3.88	12.63 ± 1.95	17.03 ± 2.87^*a*^	15.74 ± 4.03
GRN% (%)	23.14 ± 6.83	24.18 ± 19.8	20.76 ± 9.54	11.69 ± 7.33	13.54 ± 8.11	13.16 ± 8.43

^a^*p* < .05 significantly different from the AIN group.

The results of serum biochemistry (Table 8) showed that the CR levels in female rats in the GM group were higher than that in the AIN and NM groups (*p* < .05). ALT levels in male rats in the both NM and GM groups were higher than that in the AIN group (*p* < .05). Additionally, TP and GLU in male rats in the GM group were higher than those in the AIN group (*p* < .05). No significant differences were found in other indicators among the groups.

**Table 8.** Serum biochemistry in rats (*n* = 8).

	female			male		
	AIN	NM	GM	AIN	NM	GM
ALT (u/l)	19.60 ± 3.28	23.88 ± 4.43	24.20 ± 5.80	23.96 ± 2.02	33.14 ± 3.34^*a*^	30.54 ± 6.20^*a*^
AST (u/l)	141.95 ± 17.22	141.90 ± 29.27	175.38 ± 106.16	136.78 ± 31.26	155.4 ± 32.18	159.16 ± 28.28
CK (u/l)	2133.15 ± 505.45	1649.08 ± 588.29	1786.19 ± 969.54	1493.36 ± 429.49	1707.74 ± 722.18	1601.29 ± 443.56
LDH (u/l)	1859.13 ± 194.31	1927.88 ± 604.67	1958.88 ± 493.65	1968.75 ± 664.53	2222.13 ± 731.98	2527.63 ± 515.43
ALP (u/l)	50.51 ± 17.04	68.13 ± 17.26	62.14 ± 18.27	93.55 ± 21.97	89.76 ± 13.91	105.33 ± 27.69
TP (g/l)	82.68 ± 4.63	88.30 ± 3.73	87.04 ± 6.48	76.54 ± 4.54	79.58 ± 2.92	81.95 ± 3.88^*a*^
ALB (g/l)	31.84 ± 2.28	32.93 ± 4.62	33.26 ± 3.51	27.26 ± 1.67	26.73 ± 1.07	28.45 ± 1.40
TBIL (μmol/l)	1.29 ± 0.30	1.64 ± 0.99	0.94 ± 0.56	1.28 ± 0.35	1.57 ± 0.35	1.14 ± 0.32
CR (μmol/l)	30.63 ± 1.19	30.25 ± 4.03	35.88 ± 3.87^*ab*^	26.5 ± 3.16	26.38 ± 4.47	29.50 ± 4.38
BUN (mmol/l)	6.54 ± 1.07	5.28 ± 1.52	6.05 ± 0.86	5.95 ± 0.67	5.50 ± 0.55	5.74 ± 0.67
GLU (mmol/l)	4.72 ± 0.95	5.36 ± 1.16	5.46 ± 0.75	4.82 ± 0.78	5.29 ± 0.88	5.69 ± 0.49^*a*^
TC (mmol/l)	2.10 ± 0.61	2.39 ± 0.47	2.27 ± 0.42	2.42 ± 0.43	2.37 ± 0.42	2.52 ± 0.42
TG (mmol/l)	0.72 ± 0.51	0.63 ± 0.35	0.44 ± 0.29	1.30 ± 0.52	0.82 ± 0.34	1.31 ± 0.78
HDL-C (mmol/l)	0.50 ± 0.08	0.50 ± 0.07	0.51 ± 0.10	0.44 ± 0.08	0.43 ± 0.11	0.47 ± 0.10
LDL-C (mmol/l)	0.26 ± 0.11	0.35 ± 0.08	0.30 ± 0.07	0.40 ± 0.08	0.40 ± 0.07	0.40 ± 0.07

^a^*p* < .05 significantly different from the AIN group; b: *p* < .05 significantly different from the NM group.

### Organ Weight and Histopathology

As shown in Table 9, female rats in the NM and GM groups had higher brain weights than those in the AIN group (p < .05). Similarly, male rats in the NM group had higher brain and testis weights compared to the AIN group (p < .05). No significant differences were observed in the weights of other organs among the groups. In addition, there were no significant differences in the organ indexes among the three groups. Histopathological examination showed that compared with the two control groups (AIN and NM), the organs of the female and male rats in the GM group showed no obvious adverse changes (Figure S1, S2).

**Table 9.** Organ weight and organ index in rats (n = 8).

	female			male		
	AIN	NM	GM	AIN	NM	GM
brain weight (g)	1.99 ± 0.06	2.14 ± 0.04^*a*^	2.10 ± 0.07^*a*^	2.15 ± 0.08	2.30 ± 0.09^*a*^	2.26 ± 0.07
brain index (%)	0.61 ± 0.06	0.62 ± 0.05	0.63 ± 0.05	0.38 ± 0.02	0.39 ± 0.03	0.39 ± 0.04
heart weight (g)	1.14 ± 0.09	1.14 ± 0.09	1.09 ± 0.06	1.78 ± 0.25	1.77 ± 0.16	1.77 ± 0.16
heart index (%)	0.35 ± 0.03	0.33 ± 0.04	0.32 ± 0.03	0.31 ± 0.04	0.30 ± 0.02	0.30 ± 0.02
thymus weight (g)	0.34 ± 0.19	0.42 ± 0.06	0.41 ± 0.06	0.50 ± 0.09	0.50 ± 0.06	0.48 ± 0.07
thymus index (%)	0.11 ± 0.04	0.12 ± 0.02	0.12 ± 0.02	0.09 ± 0.01	0.08 ± 0.02	0.08 ± 0.01
liver weight (g)	7.81 ± 1.15	8.64 ± 0.93	8.71 ± 2.24	13.63 ± 1.78	13.58 ± 1.23	15.14 ± 2.83
liver index (%)	2.36 ± 0.22	2.50 ± 0.17	2.60 ± 0.67	2.37 ± 0.24	2.29 ± 0.12	2.55 ± 0.27
spleen weight (g)	0.58 ± 0.10	0.66 ± 0.11	0.58 ± 0.11	0.79 ± 0.17	0.87 ± 0.11	0.87 ± 0.16
spleen index (%)	0.18 ± 0.03	0.19 ± 0.03	0.17 ± 0.03	0.14 ± 0.03	0.15 ± 0.01	0.15 ± 0.02
kidney weight (g)	1.96 ± 0.15	2.06 ± 0.16	1.87 ± 0.15	3.16 ± 0.25	3.22 ± 0.30	2.93 ± 0.37
kidney index (%)	0.60 ± 0.06	0.60 ± 0.04	0.56 ± 0.05	0.55 ± 0.05	0.54 ± 0.04	0.50 ± 0.06
adrenal gland weight (g)	0.07 ± 0.02	0.07 ± 0.02	0.07 ± 0.01	0.08 ± 0.01	0.09 ± 0.02	0.08 ± 0.02
adrenal gland index (%)	0.02 ± 0.00	0.02 ± 0.01	0.02 ± 0.00	0.01 ± 0.00	0.02 ± 0.00	0.01 ± 0.00
ovary weight (g)	0.14 ± 0.05	0.14 ± 0.03	0.14 ± 0.02			
ovary index (%)	0.04 ± 0.01	0.04 ± 0.01	0.04 ± 0.01			
uterus weight (g)	0.51 ± 0.11	0.47 ± 0.09	0.57 ± 0.29			
uterus index (%)	0.16 ± 0.03	0.14 ± 0.03	0.17 ± 0.09			
testis weight (g)				3.35 ± 0.34	3.74 ± 0.22^*a*^	3.53 ± 0.24
testis index (%)				0.58 ± 0.06	0.63 ± 0.05	0.60 ± 0.06
epididymis weight (g)				1.35 ± 0.14	1.45 ± 0.15	1.38 ± 0.15
epididymis index (%)				0.24 ± 0.03	0.24 ± 0.02	0.23 ± 0.03

^a^*p* < .05 significantly different from the AIN group.

### Maroacc Gene Detection

**Figure 5.**
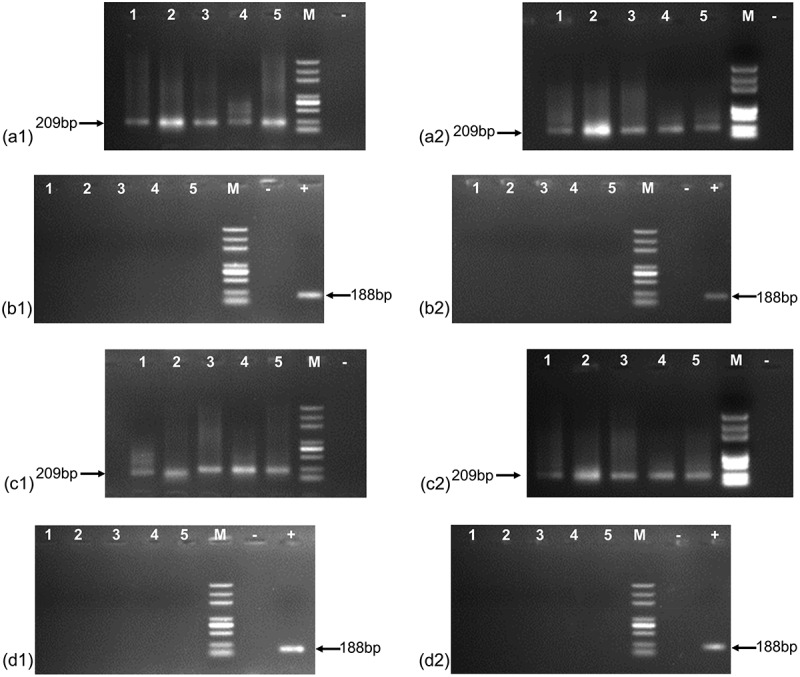
The PCR products of the β-actin and maroACC genes in main organs (a1: NM group female rat, β-actin; a2: GM group female rat, β-actin; b1: NM group female rat, maroACC; b2: GM group female rat, maroACC; c1: NM group male rat, β-actin; c2: GM group male rat, β-actin; d1: NM group male rat, maroACC; d2: GM group male rat, maroACC; M: DNA marker; -: negative control (double distilled water); +: positive control (CC-2 maize); lanes 1 to 5: brain, liver, spleen, kidney, ovary (or testis); the length of DNA marker, from top to bottom, is 5000, 3000, 2000, 1000, 750, 500, 250, 100 bp.).

Figure 5 shows that the 209 bp reference gene fragment (β-actin) was successfully amplified in the main organs of both the NM and GM groups, whereas the 188 bp maroACC gene fragment was not amplified.

## Discussion

Nowadays, the safety of GM crops attracts lots of attention of the public. Health risks of GM crops have always been a debatable topic all over the world. Researchers have done many toxicity studies to reveal relative safety of GM crops. The data analysis of a GM crop MON863 feeding study in rats suggested signs of liver and kidney toxicity,^28^ which sparked significant controversy over the use of GM crops. In another study conducted in Turkey, thirty Wistar albino rats were divided into three groups, feeding with standard rat pellet, a diet containing 20% of conventional maize or a diet containing 20% of Bt maize. After around 40 d, there were alterations in serum chemistry and hematology values.^29^ However, not all studies have reached the same conclusion. Another study of growth and health effects on rats after a 16-week of Bt maize MON 810, no adverse nutrition-related health effects were detected.^30^ Therefore, it is essential to evaluate the relative safety of GM crops cautiously.

Aside from traditional test like body weight, organ weight, hematology, etc ., we also added open-field tests, Morris water maze to evaluate the behavior, stress, spatial memory, and spatial navigation. Moreover, we monitored the offspring for 90 d to evaluate both chronic and sub-chronic toxicity.

Concerns about food safety necessitate rigorous evaluations of GM crops and their products as a part of several regulatory requirements before commercial release.^31^ This study focused on the relative safety of GR maize (CC-2) with two main objectives: to provide evidence for the safety evaluation of glyphosate-resistant transgenic crops and to fill the gap of developmental toxicity of CC-2 maize. Zhong et al.^20^ evaluated developmental toxicity of CC-2 maize by feeding it to laying hens, but only included rooster offsprings. This study aims to supplement the existing safety evaluations of CC-2 maize.

The concept of substantial equivalence is key in the safety assessment process, which is used to identify similarities and differences between the new food and their conventional counterpart.^32^ It is not a safety assessment but a starting point for structuring the safety assessment of a new food.^32,33^ The demonstration of substantial equivalence is a two-step procedure, with the first step assessing agronomic, morphological, and chemical characteristics, such as macronutrients, of the GM crops.^34^ In this study, no significant differences were found in the main components of CC-2 maize and its non-transgenic counterpart, which is consistent with the previous analyses^19,21,35^ employed integrated proteomic and metabolomic technology and found no significant unintended effects from the maroACC gene.

In numerous studies on the toxicity of GM crops on the growth and development of rats, body weight and physical development are important observational indicators. Some researches suggested that the consumption of GM crops may affect the body weight of rats,^28,36^ and body weight is a common indicator to reflect developmental toxicity. In this study, we also measured body weight. There was a difference in body weight between the two control groups (AIN and NM). Nevertheless, there were no differences between the GM group and the two control groups, which is consistent with precious 90-d feeding study in rats.^37^ This suggests that feeding CC-2 maize may has no obvious adverse effect on the rat body weight. We also took feed conversion ratio into consideration. There was a higher ratio in NM group for both male and female rats in age 6 months; however, the differences were not significant. Previous study showed that the proportion of macronutrients may affect the digestion and absorption of rats.^38^ For example, amylose may be digested more quickly compared to amylopectin. This is particular obvious as rats growing older. We did make sure the three diets were designed with theoretically equal concentrations of macronutrients. But we did not know the structure of the macronutrients. Meanwhile, the litter was used as a unit to monitor rat food intake. As a result, even though the differences were not significant, further researches are needed. Moreover, previous studies have found that the source of feed nutrients may affect the feed physical form.^39^ Meanwhile, the physical form^40^ and nutrient component^39^ of feeds could affect the body weight of experimental animals. This might explain the body weight differences between the two control groups.

Age-appropriate developmental milestones and behavioral tests can reflect the physiological and neurobehavioral development of animals.^41–43^ Our study shows that rats in the GM group and the two control groups (AIN and NM) have similar performance in various developmental milestones and behavioral tests before weaning indicating no adverse effect on the early growth and development of rats.

Open-field tests are widely used to assess anxiety, motor activity, and exploratory behaviors in experimental animals.^44,45^ Stressed rats exhibit less activity and increased active stereotyped behaviors in open spaces.^46^ Rearing, a typical exploratory behavior in tests, is negatively correlated with anxiety levels.^47^ Animals with lower anxiety tended to spend more time in the inner squares.^46,48^ In this study, male rats in the NM group showed more active exploratory behavior and lower anxiety in the open field test on PND 21 compared with the AIN group. This difference may be influenced by the environment and endocrine system^49^ and may require further exploration. No significant differences were observed in the performance between the GM group and the control groups (AIN, NM), suggesting that CC-2 maize feeding has no obvious adverse effects on exploration and anxiety.

The Morris water maze is a standard test for assessing spatial memory and spatial navigation.^50^ It usually includes a training trial and a probe trial.^51^ Because of the qualitative and quantitative differences between the developing and mature nervous systems,^52,53^ we conducted Morris water maze tests in both weaning and adult rats. There were no significant differences among groups in the two Morris water maze tests, suggesting that CC-2 maize feeding does not adversely affect on the spatial learning and memory of rats.

Blood transports nutrients and oxygen to cells and tissues, and wastes from cells and tissues; serum biochemical indicators provide specific information of various systems and organs, including metabolism, blood circulation, and fluid balance.^54^ In this study, female rats in the GM group had significantly higher CR levels than the other two control groups, while male rats showed a similar CR trend, though not significantly different. This result was similar with a 90-d feeding study of GR crops in rats except that the CR levels was statistically significant difference in male rats while female was not significantly different.^55^ Due to multiple physiologic and technological issues related to its measurement, the interpretation of serum CR requires some caution.^56^ No abnormalities were found in serum BUN, kidney weight, kidney index, and histopathology in rats. Differences in indicators were mainly between the maize feed groups (NM and GM) and the blank control group (AIN). It was considered that this may be related to the source of nutrients in the purified feed and the maize feeds. More targeted tests could be used to further explore the effect of CC-2 maize on kidney. Aside from CR, no significant differences were found between the two maize feed groups (NM and GM) indicating that CC-2 maize had no obvious adverse effects on the hematology and serum biochemistry in rats.

Organ weight and index can reflect the development and health status of organs and histopathology is the gold standard in diagnosing the presence and nature of diseases.^57^ Organ weight is greatly affected by body weight, and the organ index is a more objective and reliable measurement method that corrects the effect of body weight. This study found significant differences in the weight of individual organs between the two maize feed groups (NM and GM) and the blank control group (AIN) but no significant differences in the organ weight between the two maize feed groups. In addition, no significant differences were found in the indexes of organ. Combined with the histopathology results, no adverse changes were attributable to CC-2 maize.

Exogenous substances (genes and proteins) are crucial for evaluating GM crop food safety. At present, few studies have assessed exogenous proteins of GR crops in vivo, and the specificity of the method is still debatable. It is therefore important to assess the presence of exogenous genes in animals.^58^ In this study, primers were designed according to CC-2 insertions and side sequences to detect exogenous gene fragments. The results showed no target fragments were amplified in the main organs, which is consistent with the results of other studies of GR crops.^59^ Rapid degradation of exogenous DNA in intestinal fluid^60^ likely contributed to the negative PCR results.

## Limitation

The study has some limitations. First, differences in indicators mainly existed between the two maize feed groups (NM, GM) and the blank control group (AIN). A similar situation emerged in the study of Hu.^61^ Both this study and Hu et al.^61^ added higher doses of grain to feeds. It is speculated that the significantly differences in indicators may be related to the different nutrient sources between purified feed and grain feeds. Future experiments should include experimental and control groups with different dose levels; detailed nutritional contents of grains, such as the types of vitamins, minerals, fiber, and carbohydrates; improved the processing technology; and ensuring the consistency of the physical quality of feeds as much as possible. Second, we did not perform PCR tests on the feeds, which should be taken into account in future studies, as it ignores the fact that exogenous gene fragments may be destroyed during feed processing. Additionally, the litter was weighed as a unit to monitor food intake, the sample size was only two for male and female rats in each group. The sample size is relatively small. Therefore, even though the differences of FCR between NM and the other two groups was not significant, further studies may want to take this into consideration. Moreover, only one exogenous gene fragment was detected in this study. In addition, different loci and gene fragments sizes should be detected to provide comprehensive assessment of exogenous genes presence in animals. Last but not least, the absence of biochemical analyses (e.g., serum biochemistry, ROS, inflammatory markers) at critical early time points such as PND 21 limits the detection of potential transient alterations in specific metabolic or molecular pathways during the immediate post-weaning period. Incorporating such analyses at key developmental stages should be considered in future studies aiming to comprehensively map both developmental progress and long-term consequences of exposure.

## Conclusion

This study demonstrated that CC-2 maize is relatively safe in rats. The findings support the relative safety of glyphosate-resistant transgenic crops for growth and development. Future studies should address the identified limitations to enhance the robustness and comprehensiveness of safety evaluations for GM crops.

## Highlights

- The food safety of a GR maize was assessed by feeding pregnant and filial rats.
- The feed formulae were based on the principle of maximum carbohydrate addition.
- CC-2 maize is as safe as receptor maize for growth and development in rats.

## Supplementary Material

Supplementary.docx

## References

[1] Zhang C, Wohlhueter R, Zhang H. Genetically modified foods: a critical review of their promise and problems. Food Sci Hum Wellness. 2016;5(3):116–23. doi: 10.1016/j.fshw.2016.04.002.

[2] Raman R. The impact of genetically modified (GM) crops in modern agriculture: a review. GM Crops Food. 2017 Oct 2. 8(4):195–208. doi: 10.1080/21645698.2017.1413522. 29235937 PMC5790416

[3] Delaney B, Goodman RE, Ladics GS. Food and feed safety of genetically engineered food crops. Toxicol Sci. 2018 Apr 1. 162(2):361–71. doi: 10.1093/toxsci/kfx249. 29211881

[4] Heap I, Duke SO. Overview of glyphosate-resistant weeds worldwide. Pest Manag Sci. 2018 May. 74(5):1040–49. doi: 10.1002/ps.4760. 29024306

[5] Duke SO. The history and current status of glyphosate. Pest Manag Sci. 2018 May. 74(5):1027–34. doi: 10.1002/ps.4652. 28643882

[6] Green JM, Siehl DL. History and outlook for glyphosate-resistant crops. Rev Environ Contam Toxicol. 2021;255:67–91. 34109481 10.1007/398_2020_54

[7] Gatew H, Mengistu K. Genetically modified foods (GMOs); a review of genetic engineering. J Life Sci Biomed. 2019 Nov 25. 9(6):157–63. doi: 10.36380/scil.2019.jlsb25.

[8] König A, Cockburn A, Crevel RWR, Debruyne E, Grafstroem R, Hammerling U, Kimber I, Knudsen I, Kuiper HA, Peijnenburg AACM, et al. Assessment of the safety of foods derived from genetically modified (GM) crops. Food Chem Toxicol. 2004 Jul 1. 42(7):1047–88. doi: 10.1016/j.fct.2004.02.019. 15123382

[9] Salisu IB, Shahid AA, Ali Q, Rao AQ, Husnain T. Nutritional assessment of dietary Bt and Cp4EPSPS proteins on the serum biochemical changes of rabbits at different developmental stages. Front Nutr. 2018;5:49. doi: 10.3389/fnut.2018.00049. 29922652 PMC5996157

[10] Steinberg P, van der Voet H, Goedhart PW, Kleter G, Kok EJ, Pla M, Nadal A, Zeljenková D, Aláčová R, Babincová J, et al. Lack of adverse effects in subchronic and chronic toxicity/carcinogenicity studies on the glyphosate-resistant genetically modified maize NK603 in Wistar Han RCC rats. Arch Toxicol. 2019 Apr. 93(4):1095–139. doi: 10.1007/s00204-019-02400-1. 30756133 PMC7261740

[11] Zhao H, Song W, Lai J. Cloning of Sorghum bicolor chloroplast transit peptide (CTP) of 5-enolpyruvylshikimate-3-phosphate synthase (EPSPS) and its functional validation in transgenic maize (Zea mays). J Agric Biotechnol Chin. 2013;21(9):1009–18.

[12] Team C. ChemLinked. [Gb 15193.29-2020 national food safety standard extended one-generation reproductive toxicity test. 2025 May 10. http://food.chemlinked.com/database/view/2206.

[13] Team C. ChemLinked. GB 15193.13-2015 national food safety standard 90 days oral toxicity test. 2025 May 10. http://food.chemlinked.com/database/view/2576.

[14] OECD [Internet]. Test no.443: Extended one-generation reproductive toxicity study. 2018 [cited 2025 May 11]. https://www.oecd.org/en/publications/test-no-443-extended-one-generation-reproductive-toxicity-study_9789264185371-en.html.

[15] OECD [Internet]. Test no. 426: developmental neurotoxicity study. 2007 [cited 2025 May 11]. https://www.oecd.org/en/publications/test-no-426-developmental-neurotoxicity-study_9789264067394-en.html.

[16] United States Environmental Protection Agency. Health Effects Test Guidelines OPPTS 870.3700 Prenatal Developmental Toxicity Study (EPA 712–C–98–207). U.S. Government Printing Office. 1998 Aug. https://www.epa.gov/epahome/research.htm

[17] Zhong R, Zhang L, Chen L, Yang X, Zhang H. Modulation of cecal microbiota in laying hens via intake of genetically modified corn with the maroACC or mCry1ac genes. J Sci Food Agric. 2020 Dec. 100(15):5450–57. doi: 10.1002/jsfa.10596. 32562272

[18] Chen L, Zhong R, Zhang L, Zhang H. The chronic effect of transgenic maize line with mCry1ac or maroACC gene on ileal microbiota using a hen model. Microorganisms. 2019 Mar. 7(3):92. doi: 10.3390/microorganisms7030092. 30909622 PMC6463162

[19] Zhong RQ, Chen L, Gao LX, Zhang LL, Yao B, Yang XG, Zhang HF. Effects of feeding transgenic corn with mCry1ac or maroACC gene to laying hens for 12 weeks on growth, egg quality and organ health. Animal. 2016 Aug. 10(8):1280–87. doi: 10.1017/S1751731116000203. 26915544

[20] Zhong R, Chen L, Zhang L, Zhang H. The transgenerational effect of feeding genetically modified maroACC corn to laying hens and offspring roosters on offspring roosters growth and reproduction. J Anim Sci. 2017 Aug 1. 95(suppl_4):188–89. doi: 10.2527/asasann.2017.380.

[21] Zhong R, Chen L, Gao L, Zhang L, Zhang H. Evaluation of the compositional and nutritional values of mCry1Ac corn and maroACC corn in growing pigs. J Anim Sci. 2017 Aug 1. 95(suppl_4):343–44. doi: 10.2527/asasann.2017.705.

[22] Authority EFS. Explanatory statement for the applicability of the guidance of the EFSA Scientific Committee on conducting repeated-dose 90-day oral toxicity study in rodents on whole food/feed for GMO risk assessment. Efsa J. 2014;12(10):3871. 10.2903/j.efsa.2014.3871 PMC13136980 42087934

[23] Melo-Durán D, Perez JF, González-Ortiz G, Villagómez-Estrada S, Bedford MR, Graham H, Sola-Oriol D. Growth performance and total tract digestibility in broiler chickens fed different corn hybrids. Poult Sci. 2021 Aug. 100(8):101218. doi: 10.1016/j.psj.2021.101218. 34198097 PMC8255229

[24] Geerlofs L, He Z, Xiao S, Xiao ZC. 15-day subchronic developmental toxicity studies of ursolic acid in rats. Food Chem Toxicol. 2020 Oct. 144:111537. doi: 10.1016/j.fct.2020.111537. 32649969

[25] Festing MF. On determining sample size in experiments involving laboratory animals. Lab Anim. 2018 Aug. 52(4):341–50. doi: 10.1177/0023677217738268. 29310487

[26] Matei-Lațiu MC, Gal AF, Rus V, Buza V, Martonos C, Lațiu C, Ștefănuț L-C. Intestinal dysbiosis in rats: interaction between amoxicillin and probiotics, a histological and immunohistochemical evaluation. Nutrients. 2023 Feb 23. 15(5):1105. doi: 10.3390/nu15051105. 36904107 PMC10004829

[27] Bethi CMS, Jayprakash G, Muthukumar SP, Kudre TG. Application of proteins from different meat processing wastewater streams as a dietary protein source in animal feed. J Environ Manage. 2021 Dec 1. 299:113662. doi: 10.1016/j.jenvman.2021.113662. 34492438

[28] Séralini GE, Cellier D, De Vendomois JS. New analysis of a rat feeding study with a genetically modified maize reveals signs of hepatorenal toxicity. Arch Environ Contam Toxicol. 2007 May. 52(4):596–602. doi: 10.1007/s00244-006-0149-5. 17356802

[29] Kılıçgün H, Gursul C, Gökşen G, Sunar M. The comparative effects of genetically modified maize and conventional maize on rats. J Clin Anal Med. 2013 Mar 1. 4(2):136–39. doi: 10.4328/JCAM.983.

[30] Szymczyk B, Szczurek W, Swiatkiewicz S, Krzysztof K, Sieradzki Z, Mazur M, Bednarek D, Reichert M. Results of a 16-week safety assurance study with rats fed genetically modified Bt maize: effect on growth and health parameters. J Vet Res [Internet]. 2018 [cited 2025 Aug 13]. 62(4). 555–61. https://www.researchgate.net/publication/329014919_Results_of_a_16-week_Safety_Assurance_Study_with_Rats_Fed_Genetically_Modified_Bt_Maize_Effect_on_Growth_and_Health_Parameters. 30729216 10.2478/jvetres-2018-0060 PMC6364163

[31] Giraldo PA, Shinozuka H, Spangenberg GC, Cogan NOI, Smith KF. Safety assessment of genetically modified feed: is there any difference from food? Front Plant Sci. 2019;10:1592. doi: 10.3389/fpls.2019.01592. 31921242 PMC6918800

[32] Cac/gl 45-2003 guideline for the conduct of food safety assessment of foods derived from recombinant-CAC standards-international-food laws & regulations-documents-global FoodMate [Internet]. [2025 May 11]. http://files.foodmate.com/2013/files_1782.html.

[33] Division AP and H. GM food safety assessment tools for trainers. 2009 [cited 2025 May 11]; https://openknowledge.fao.org/handle/20.500.14283/i0110e.

[34] Zetterberg C, Edvardsson Björnberg K. Time for a new EU regulatory framework for GM crops? J Agric Environ Ethics. 2017 June 1. 30(3):325–47. doi: 10.1007/s10806-017-9664-9.

[35] Liu W, Zhao H, Miao C, Jin W. Integrated proteomics and metabolomics analysis of transgenic and gene-stacked maize line seeds. GM Crops Food. 2021 Jan 2. 12(1):361–75. doi: 10.1080/21645698.2021.1934351. 34097556 PMC8189116

[36] Han S, Zou S, He X, Huang K, Mei X. Potential subchronic food safety of the stacked trait transgenic maize GH5112E-117C in Sprague-Dawley rats. Transgenic Res. 2016 Feb 26. 25(4):453–63. doi: 10.1007/s11248-016-9944-6. 26919987

[37] Chen C, Shi L, Mao H, Han C, Zhao J, Zhuo Q, Li Y. Safety assessment of transgenic maize CC-2 by 90-day feeding study in Sprague-Dawley rats. Toxicol Res. 2024 Mar 5. 13(2):tfae025. doi: 10.1093/toxres/tfae025. PMC10939339 38496381

[38] Garrison MV, Breidenstein CP. Digestion of sugarcane by the Polynesian rat. J Wildl Manag. 1970;34(3):520–22. doi: 10.2307/3798856.

[39] Amoozmehr A, Dastar B, Ashayerizadeh O, Mirshekar R, Abdollahi MR. Effect of feed form and nutrient density on growth performance, blood parameters, and intestinal traits in broiler breeder pullets. Poult Sci. 2023 Jul 1. 102(7):102700. doi: 10.1016/j.psj.2023.102700. 37141808 PMC10311152

[40] Malheiros JM, Correia BSB, Ceribeli C, Bruscadin JJ, Diniz WJS, Banerjee P, da Silva Vieira D, Cardoso TF, Andrade BGN, Petrini J, et al. Ruminal and feces metabolites associated with feed efficiency, water intake and methane emission in Nelore bulls. Sci Rep. 2023 Oct 21. 13(1):18001. doi: 10.1038/s41598-023-45330-w. 37865691 PMC10590413

[41] Yang R, Liu S, Zheng Y, Zhang M, Dang R, Tang M. Maternal diet of polyunsaturated fatty acid influence the physical and neurobehaviour of rat offspring. Int J Dev Neurosci. 2018 Dec. 71(1):156–62. doi: 10.1016/j.ijdevneu.2018.09.005. 30223009

[42] Moore CL, Flanigan TJ, Law CD, Loukotková L, Woodling KA, da Costa GG, Fitzpatrick SC, Ferguson SA. Developmental neurotoxicity of inorganic arsenic exposure in Sprague-Dawley rats. Neurotoxicol Teratol. 2019;72:49–57. doi: 10.1016/j.ntt.2019.01.007. 30738146

[43] Rüedi-Bettschen D, Platt DM. Detrimental effects of self-administered methamphetamine during pregnancy on offspring development in the rat. Drug Alcohol Depend. 2017 Aug 1. 177:171–77. doi: 10.1016/j.drugalcdep.2017.03.042. 28600929 PMC5701573

[44] Poveda CM, Popović N, Morales-Delgado N, la cruz-Sánchez E D, Caballero Bleda M, Popović M. The diurnal variation of open-field habituation in rats. Behav Processes. 2020 Sep. 178:104186. doi: 10.1016/j.beproc.2020.104186. 32619522

[45] Knight P, Chellian R, Wilson R, Behnood-Rod A, Panunzio S, Bruijnzeel AW. Sex differences in the elevated plus-maze test and large open field test in adult Wistar rats. Pharmacol Biochem Behav. 2021 May. 204:173168. doi: 10.1016/j.pbb.2021.173168. 33684454 PMC8130853

[46] Kraeuter AK, Guest PC, Sarnyai Z. The open field test for measuring locomotor activity and anxiety-like behavior. Methods Mol Biol. 2019;1916:99–103. 30535687 10.1007/978-1-4939-8994-2_9

[47] Sturman O, Germain PL, Bohacek J. Exploratory rearing: a context- and stress-sensitive behavior recorded in the open-field test. Stress. 2018 Sep. 21(5):443–52. doi: 10.1080/10253890.2018.1438405. 29451062

[48] Padurariu M, Antioch I, Balmus I, Ciobica A, El-Lethey HS, Kamel MM. Describing some behavioural animal models of anxiety and their mechanistics with special reference to oxidative stress and oxytocin relevance. Int J Vet Sci Med. 2017 Dec. 5(2):98–104. doi: 10.1016/j.ijvsm.2017.08.003. 30255057 PMC6137856

[49] Kentner AC, Lima E, Migliore MM, Shin J, Scalia S. Complex environmental rearing enhances social salience and affects hippocampal corticotropin releasing hormone receptor expression in a sex-specific manner. Neuroscience. 2018 Jan 15. 369:399–411. doi: 10.1016/j.neuroscience.2017.11.035. 29183827

[50] Morris RG, Garrud P, Rawlins JN, O’Keefe J. Place navigation impaired in rats with hippocampal lesions. Nature. 1982 June 24. 297(5868):681–83. doi: 10.1038/297681a0. 7088155

[51] Tomás Pereira I, Burwell RD. Using the spatial learning index to evaluate performance on the water maze. Behav Neurosci. 2015 Aug. 129(4):533–39. doi: 10.1037/bne0000078. 26214218 PMC5077721

[52] Barone S, Das KP, Lassiter TL, White LD. Vulnerable processes of nervous system development: a review of markers and methods. Neurotoxicology. 2000;21(1–2):15–36. 10794382

[53] Barnhart CD, Yang D, Lein PJ. Using the Morris water maze to assess spatial learning and memory in weanling mice. PLOS ONE. 2015;10(4):e0124521. doi: 10.1371/journal.pone.0124521. 25886563 PMC4401674

[54] Caceres PS, Benedicto I, Lehmann GL, Rodriguez-Boulan EJ. Directional fluid transport across organ–blood barriers: physiology and cell biology. Cold Spring Harb Perspect Biol. 2017 Mar. 9(3):a027847. doi: 10.1101/cshperspect.a027847. 28003183 PMC5334253

[55] Zhu Y, He X, Luo Y, Zou S, Zhou X, Huang K, Xu W. A 90-day feeding study of glyphosate-tolerant maize with the G2-aroA gene in Sprague-Dawley rats. Food Chem Toxicol. 2013 Jan 1. 51:280–87. doi: 10.1016/j.fct.2012.09.008. 23000447

[56] Kashani K, Rosner MH, Ostermann M. Creatinine: from physiology to clinical application. Eur J Intern Med. 2020 Feb. 72:9–14. doi: 10.1016/j.ejim.2019.10.025. 31708357

[57] Angel Arul Jothi J, Mary Anita Rajam V. A survey on automated cancer diagnosis from histopathology images. Artif Intell Rev. 2017 June 1. 48(1):31–81. doi: 10.1007/s10462-016-9494-6.

[58] Shahid AA, Salisu IB, Yaqoob A, Rao AQ, Ullah I, Husnain T. Assessing the fate of recombinant plant DNA in rabbit’s tissues fed genetically modified cotton. J Anim Physiol Anim Nutr. 2020 Jan. 104(1):343–51. doi: 10.1111/jpn.13243. 31701592

[59] Long L, Zhao N, Li C, He Y, Dong L, Yan W, Xing Z, Xia W, Ma Y, Xie Y, et al. Development and collaborative validation of an event-specific quantitative real-time PCR method for detection of genetically modified CC-2 maize. Front Plant Sci. 2024;15:1460038. doi: 10.3389/fpls.2024.1460038. 39319004 PMC11420048

[60] Xing R, Liu H, Qi X, Pan L. Measuring the process and rate of exogenous DNA degradation during digestion in mice. Sci Rep. 2022 Apr 19. 12(1):6463. doi: 10.1038/s41598-022-10340-7. 35440601 PMC9018913

[61] Hu Y, Guo M, Zhuo Q, Han C, Shi L, Mao H, Li Y, Zhao J, Chen C, Yang X. Three-generation reproductive toxicity of genetically modified maize with Cry1Ab and EPSPS genes in rats. J Agric Food Chem. 2020 Sep 30. 68(39):10912–19. doi: 10.1021/acs.jafc.0c02237. 32649186

