# Supplementary material for: Relative safety of glyphosate-resistant maize (CC-2) in rats
Source: GM Crops Food. 2025 Sep 12;16(1):591–606. doi: 10.1080/21645698.2025.2550820 (PMC12439554; doi:10.1080/21645698.2025.2550820)
Supplement: Supplementary.docx [file KGMC_A_2550820_SM7835.docx]

Table S1 Body weight in rats (before weaning, n=16; after weaning, n=8)

| age | female | | |  | male | | |
| --- | --- | --- | --- | --- | --- | --- | --- |
|  | AIN | NM | GM |  | AIN | NM | GM |
| During lactation | |  |  |  |  |  |  |
| 4d | 11.39±2.33 | 11.60±0.88 | 11.97±1.11 |  | 11.96±2.14 | 12.17±1.04 | 12.34±1.00 |
| 7d | 19.32±2.47 | 19.35±1.18 | 19.75±1.26 |  | 19.94±2.45 | 20.09±1.51 | 20.36±0.99 |
| 14d | 38.38±3.96 | 39.82±2.28 | 38.86±1.96 |  | 39.95±4.08 | 40.76±2.30 | 39.70±1.82 |
| 21d | 65.04±6.43 | 64.90±3.39 | 63.02±3.40 |  | 67.71±7.23 | 66.92±3.33 | 64.60±4.52 |
| After weaning | |  |  |  |  |  |  |
| 3w | 62.19±2.05 | 65.44±2.96 | 63.19±3.25 |  | 64.06±3.43 | 66.44±2.72 | 64.94±3.25 |
| 4w | 97.44±5.03 | 102.63±5.97 | 102.13±5.62 |  | 102.63±5.10 | 111.31±7.13^a^ | 106.50±6.42 |
| 5w | 142.06±8.84 | 141.63±8.55 | 148.69±6.92 |  | 161.19±6.31 | 173.75±11.67^a^ | 166.88±9.08 |
| 6w | 182.81±13.45 | 189.63±6.25 | 188.00±15.09 |  | 231.00±11.72 | 246.06±16.93 | 232.44±15.44 |
| 7w | 217.38±18.26 | 223.44±12.75 | 221.19±14.23 |  | 304.50±19.27 | 312.25±21.75 | 286.13±30.08 |
| 8w | 244.56±22.28 | 245.13±25.43 | 251.06±15.39 |  | 366.63±27.35 | 374.00±22.27 | 350.63±40.33 |
| 9w | 259.56±24.88 | 272.00±40.74 | 277.94±12.23 |  | 416.94±39.69 | 446.56±19.83 | 408.13±39.54 |
| 10w | 280.31±32.29 | 301.88±33.57 | 283.81±26.57 |  | 434.56±54.96 | 504.06±20.40^a^ | 453.25±39.75 |
| 11w | 283.69±45.49 | 319.75±29.98 | 294.00±34.81 |  | 462.38±43.24 | 519.75±41.40 | 485.81±48.79 |
| 12w | 296.56±42.09 | 340.50±27.02 | 297.00±37.56 |  | 493.38±39.82 | 524.63±81.45 | 510.13±52.84 |
| 13w | 306.25±40.05 | 346.19±27.85 | 308.81±35.02 |  | 511.25±40.88 | 535.00±88.30 | 541.50±52.42 |
| 14w | 316.31±37.32 | 355.19±29.70 | 326.63±37.17 |  | 539.31±42.79 | 551.31±73.48 | 555.38±56.23 |
| 15w | 323.81±38.54 | 364.31±28.25 | 335.13±40.55 |  | 565.56±39.94 | 582.50±59.06 | 587.81±54.73 |
| 16w | 328.44±33.45 | 347.00±27.59 | 335.56±36.72 |  | 566.25±39.54 | 588.81±53.89 | 587.56±59.32 |

*a*: P < 0.05 significantly different from the AIN group.

Table S2 Feed conversion ratio with different feeds

|  | 4 month | 5 month | 6 month | Total |
| --- | --- | --- | --- | --- |
| Female |  |  |  |  |
| AIN | 2.80±0.24 | 6.93±0.86 | 11.85±1.07 | 5.34±0.13 |
| NM | 2.76±0.27 | 6.63±1.57 | 22.21±5.24 | 5.91±0.59 |
| GM | 2.67±0.04 | 8.04±2.00 | 13.73±2.44 | 5.63±0.18 |
| Male |  |  |  |  |
| AIN | 1.99±0.04 | 3.82±0.37 | 7.57±0.54 | 3.71±0.16 |
| NM | 2.26±0.15 | 3.98±0.48 | 10.22±1.97 | 3.95±0.08 |
| GM | 2.20±0.11 | 4.00±0.50 | 8.11±0.50 | 4.03±0.12 |


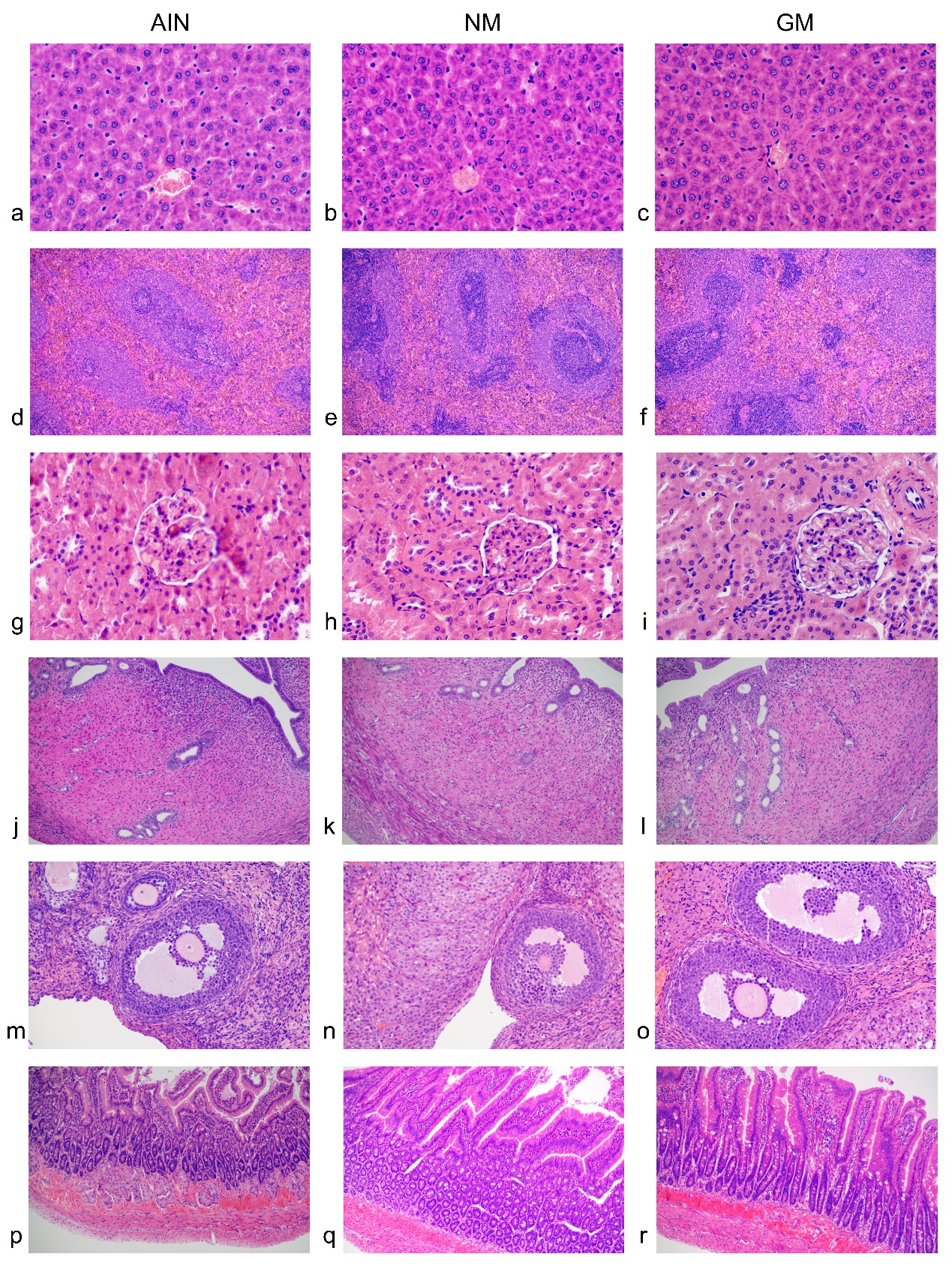


Figure S1 Histopathological examination of female rat organs ( H & E, Liver(a-c)×400, spleen(d-f) ×100, kidney(g-i)×400, uterus (j-l)×100, ovary(m-o)×200, duodenum(p-r)×100)


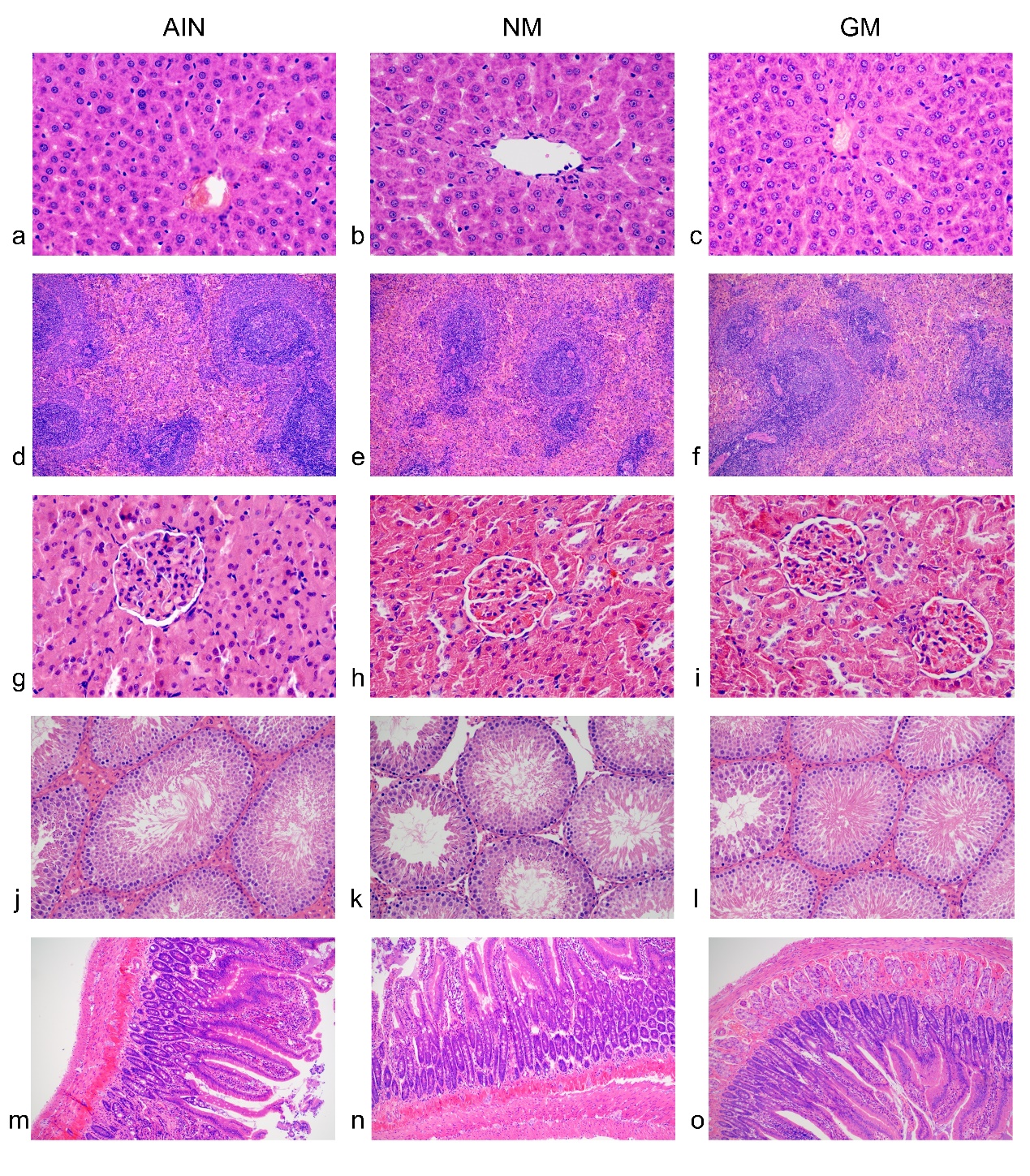


Figure S2 Histopathological examination of male rat organs ( H & E, Liver(a-c)×400, spleen(d-f) ×100, kidney(g-i)×400, testis(j-l)×200, duodenum(m-o)×100)
